# Extracellular acidification stimulates GPR68 mediated IL-8 production in human pancreatic β cells

**DOI:** 10.1038/srep25765

**Published:** 2016-05-11

**Authors:** Vikash Chandra, Angeliki Karamitri, Paul Richards, Françoise Cormier, Cyrille Ramond, Ralf Jockers, Mathieu Armanet, Olivier Albagli-Curiel, Raphael Scharfmann

**Affiliations:** 1 INSERM, U1016, Institut Cochin, Université Paris Descartes, Sorbonne Paris Cité, Faculté de Médecine, Paris, 75014, France; 2 Cell Therapy Unit, Hôpital Saint Louis, AP-HP, and University Paris-Diderot, Paris, 75010, France

## Abstract

Acute or chronic metabolic complications such as diabetic ketoacidosis are often associated with extracellular acidification and pancreatic β-cell dysfunction. However, the mechanisms by which human β-cells sense and respond to acidic pH remain elusive. In this study, using the recently developed human β-cell line EndoC-βH2, we demonstrate that β-cells respond to extracellular acidification through GPR68, which is the predominant proton sensing receptor of human β-cells. Using gain- and loss-of-function studies, we provide evidence that the β-cell enriched transcription factor RFX6 is a major regulator of GPR68. Further, we show that acidic pH stimulates the production and secretion of the chemokine IL-8 by β-cells through NF-кB activation. Blocking of GPR68 or NF-кB activity severely attenuated acidification induced IL-8 production. Thus, we provide mechanistic insights into GPR68 mediated β-cell response to acidic microenvironment, which could be a new target to protect β-cell against acidosis induced inflammation.

In biological systems, cells actively partake in maintaining homeostasis of their environmental milieu within a precise range of physiological parameters. Cellular systems also foster the unique ability to respond and adapt to physiological stress, preserving survival and function. Signal transduction across cell membrane, through surface receptors is fundamental to detect and respond to changes in the local milieu^1^. Protons (H^+^) represent an important component of the extracellular milieu^2^. The extracellular fluids and blood pH are tightly regulated and maintained judiciously at ~7.4 but under many patho-physiological circumstances such as inflammation, ischemia and tumor formation, acidosis occurs in the localized microenvironment^3^.

Cells sense extracellular protons concentration by a number of mechanisms^4^,^5^. Ion channels such as transient receptor potential V1 and acid-sensing ion channels (ASICs) represent one sensing mechanism. Such channels are predominantly expressed on sensory neurons and act as proton sensors for pain and nociception signals^6^,^7^. A sub-family of G protein-coupled receptors (GPCR) represents a second type of proton sensing mechanism. This includes four members: GPR4, GPR68 (or Ovarian cancer G protein-couple receptor 1, OGR1), GPR65 (or T-cell death-associated gene 8, TDAG8) and GPR132 (or G2A). These receptors sense moderate extracellular pH within a narrow range (pH 6.0 to 7.6) and signal via a variety of intracellular pathways. For example, GPR68 is coupled to the G_q/11_-phospholipase-C/Ca^2+^ pathway, whereas GPR4 and GPR65 are coupled to the G_s_-adenyl-cyclase/cAMP pathway^8^,^9^.

Insulin-producing pancreatic β-cells are highly differentiated cells that play a critical role in maintaining glucose homeostasis. They are factories dedicated to produce and secrete insulin in a tightly regulated fashion^10^. β-cells sense a myriad of circulating factors such as glucose, neurotransmitters and hormones that regulate their function under physiological conditions^11^. They are also sensitive to inflammatory cytokines that are implicated in their destruction in type 1 diabetes (T1D)^12^,^13^. A recurring complication of T1D is diabetic ketoacidosis (DKA) resulting in ketonemia and metabolic acidosis^14^ with extracellular acidification of the pancreatic microenvironment^15^,^16^. However, the mechanism by which human β-cells sense proton concentration and transmit their signal remains largely unknown. It is likely that moderate acidosis in the pancreatic microenvironment is primarily sensed through the proton sensing GPCR because i) ASICs ion channels are not reported to be present in islets,^17^,^18^ ii) TRPV1 channels, even though reported to be expressed in some β cell-lines, sense acidic pH (pH 4–5)^17^,^19^,^20^,^21^. Information is limited on the expression and function of proton sensing GPCRs in pancreatic β-cells. Impaired glucose-stimulated insulin secretion has been described in GPR68 knockout mice, however the role of proton sensing GPCRs in human β-cells remains to be explored^22^.

Here, we provide evidence that GPR68 is the predominant proton sensing receptor expressed by human β-cells. Its expression is tightly regulated by RFX6, a β-cell enriched transcription factor^23^. We also show using the human β cell line Endo-CβH2^24^ that extracellular acidification activates GPR68, inducing the production and secretion of the chemokine IL-8 through NF-кB activation. In conclusion, proton sensing via GPR68 is a novel mechanism for the induction of inflammatory response in human pancreatic β-cell.

## Results

### The proton-sensing receptor GPR68, a target of RFX6, is expressed in EndoC-βH2 cells and human islets

**Figure 1:**
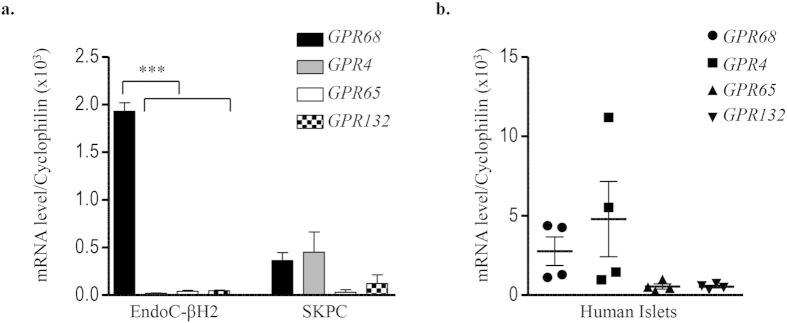
Expression of proton sensing GPCRs in EndoC-βH2, SKPC and human islets. Transcript levels of proton sensing GPCRs (*GPR68, GPR4, GPR65* and *GPR132*) determined by RT-qPCR in (**a**) EndoC-βH2 cells compared with ductal carcinoma SKPC cell-line; (**b**) human islet preparation. Data represented as mean values ± SEM of at least 3 independent experiments. ***p < 0.001; (one-way ANOVA, followed by a Tukey’s multiple comparisons post-test).

Our previously published transcriptomic analyses (GEO No: GSE48101) indicated that EndoC-βH2 cells express mRNA coding for the proton-sensing receptor *GPR68*^24^. We validated these data by Real-Time-quantitative PCR (RT-qPCR) that indicated that *GPR68* mRNA expression was enriched in EndoC-βH2 cells compared to the duct cell line SKPC (Fig. 1a). Transient transfection of EGFP tagged human GPR68 construct in EndoC-βH2 cells showed its predominant localization on the plasma membrane (Supplementary Fig. 1). GPR68 was almost the sole proton sensing GPCR expressed in EndoC-βH2 cells, the other ones (*GPR4, GPR65, GPR132*) being expressed at nearly undetectable levels (Fig. 1a). Similar data were obtained using human islet preparations that expressed *GPR68*, but not *GPR65* and *GPR132* (Fig. 1b). Of note, *GPR4* was detected in human islets and not in EndoC-βH2 cells (Fig. 1a), which could be due to its expression by non β-cells present in human islet preparations like endothelial cells^25^,^26^.

**Figure 2:**
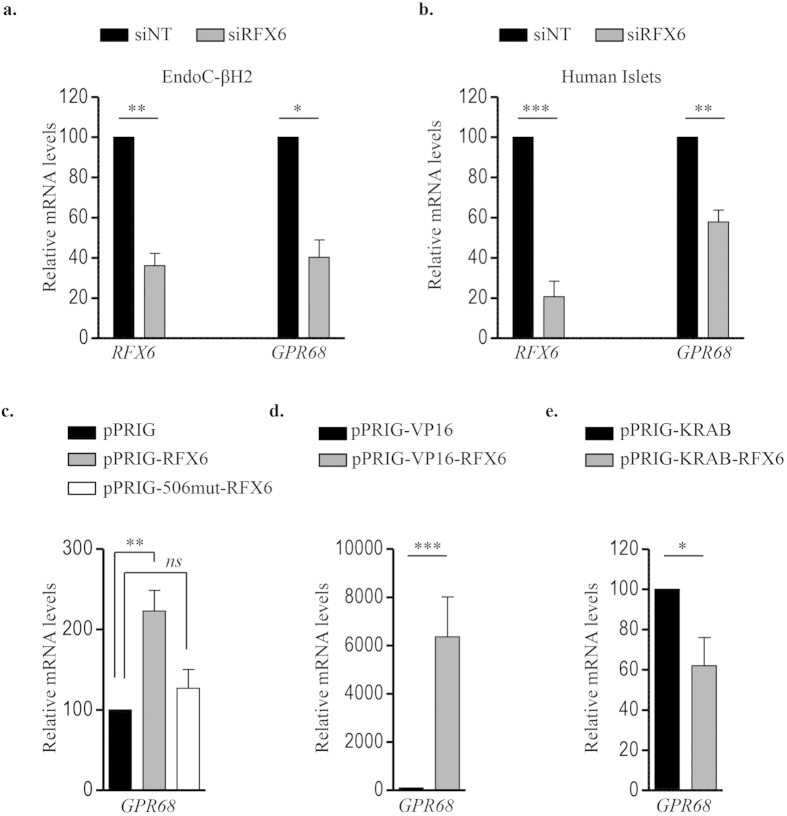
RFX6 regulates GPR68 expression in human β-cells. EndoC-βH2 cells (**a**) and adult human islets (**b**) were transfected with control non-target siRNA (siNT) or siRNA targeting *RFX6* (siRFX6). *GPR68* expression was analyzed 72 h post transfection by RT-qPCR. Data are expressed as percentage of siNT transfected cells. **(c–e)** wtRFX6 and Mut506RFX6 (one-way ANOVA) (**c**), transactivation domain VP16- conjugated RFX6 (**d**) and transcriptional repression domain KRAB- conjugated RFX6 (**e**) were expressed in EndoC-βH2 cells using bicistronic constructs with IRES-EGFP. GFP^+ve^ cells were FACS isolated 48 h post-transfection and *GPR68* expression was analyzed by RT-qPCR. Data are mean ± SEM of 3–5 experiments. *p < 0.05; **p < 0.01; ***p < 0.001 and *ns*, not significant.

RFX6 is a key transcription factor highly expressed in β-cells and required for their function. Our previous transcriptomic analyses indicated that siRNA-mediated RFX6 knock-down decreased GPR68 expression in EndoC-βH2 cells [FC, −3.85; *p* = 7.78.10^−5^] (GEO No: GSE59049) without effecting the expression of other proton sensing receptors^23^. Further validation by RT-qPCR showed that decreased expression level of *RFX6* mRNA (63.92 ± 10.5%) was consistently accompanied by decrease in the level of *GPR68* mRNA (59.73 ± 15%) in EndoC-βH2 cells (Fig. 2a). Similar results were obtained in human islets where decreased *RFX6* expression (79.34 ± 13%) resulted in a 42.15 ± 10% decrease of *GPR68* transcripts (Fig. 2b). Additionally, overexpression of wtRFX6 but not p.V506G mutant RFX6^23^, increased the expression of *GPR68* transcripts (Fig. 2c). *GPR68* expression was also enhanced following transfection of EndoC-βH2 cells with a trans-activation domain VP16 conjugated RFX6 (Fig. 2d), while transfection of a trans-repression domain conjugated KRAB-RFX6 in EndoC-βH2 cells resulted in a decreased expression of *GPR68* (Fig. 2e). GPR68 is thus the major proton sensor expressed in β-cells, its expression being tightly controlled by RFX6.

### GPR68 is involved in proton-induced inositol phosphate (IP) production in Human β-cells

**Figure 3:**
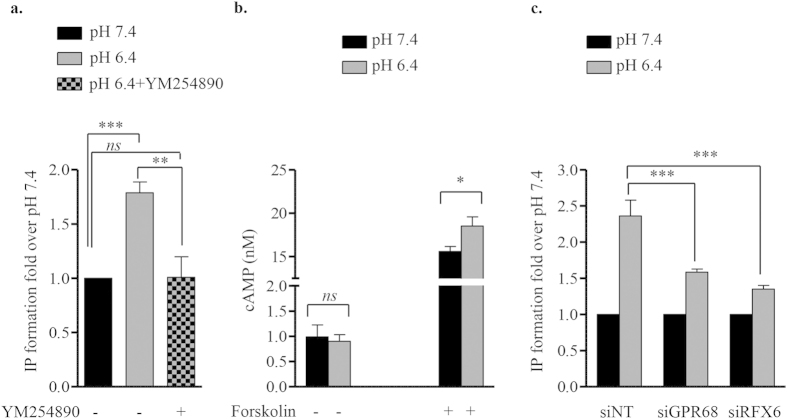
Extracellular pH modulates IP production but not cAMP accumulation in EndoC-βH2 cells. (**a**) IP formation was determined in EndoC-βH2 cells incubated at pH 7.4 and 6.4. The G_q/11_-selective inhibitor compound YM-254890 (100 nM) was also tested. Results are expressed as fold change over the IP values at pH 7.4. (**b**) For cAMP formation assay, EndoC-βH2 cells were incubated for 30 min with and without Forskolin (25 μM) at pH 7.4 or 6.4. Results are expressed as cAMP accumulation (nM) per 5,000 cells. **(c)** EndoC-βH2 cells were transfected with control siRNA (siNT), siRNA targeting either *GPR68* (siGPR68) or *RFX6* (siRFX6). 72 h post transfection, cells were incubated at pH 7.4 or 6.4 for 30 min and analyzed for the IP formation. Results are expressed as fold change over the IP values at pH 7.4. Data are mean ± SEM of 3–5 experiments. *p < 0.05; **p < 0.01; ***p < 0.001 and *ns*, not significant (one-way ANOVA, followed by a Tukey’s multiple comparisons post-test).

GPR68 is a proton-sensing G_q/11_ coupled receptor that stimulates IP formation to elicit pH dependent responses^8^. To examine if acidification of the extracellular medium activates G_q/11_ pathway in EndoC-βH2 cells, we incubated cells at either physiological pH 7.4 or acidic pH 6.4. Buffered pH media did not alter cell morphology, viability (Supplementary Fig. 2a–c) or insulin secretion in EndoC-βH2 cells (Supplementary Fig. 3). Acidic pH 6.4 induced a significant increase in IP formation (Fig. 3a). This effect was blocked by YM-254890, a selective G_q/11_ inhibitor^27^, demonstrating the selective role of G_q/11_ pathway in pH dependent responses in EndoC-βH2 (Fig. 3a). On the other hand, proton did not modulate cAMP production measured at pH 7.4 or 6.4 (Fig. 3b), further indicating that G_s_-coupled receptors such as GPR4 and GPR132 are not involved in proton sensing in EndoC-βH2 cells. We next show that acidic pH-stimulated IP production was GPR68-dependent. Indeed control (siNT) treated β-cells sensed normally the extracellular acidic pH (6.4) and responded by increasing IP formation. In contrast siRNA-mediated GPR68 depletion significantly decreased this induction (Fig. 3c). Accordingly, siRFX6 treatment lead to decreased *GPR68* expression also inhibited proton induced IP formation (Fig. 3c). Thus, GPR68 is involved in proton-induced IP production in EndoC-βH2 cells.

### EndoC-βH2 cells express and secrete the pro-inflammatory cytokine IL-8 upon exposure to acidic pH

**Figure 4:**
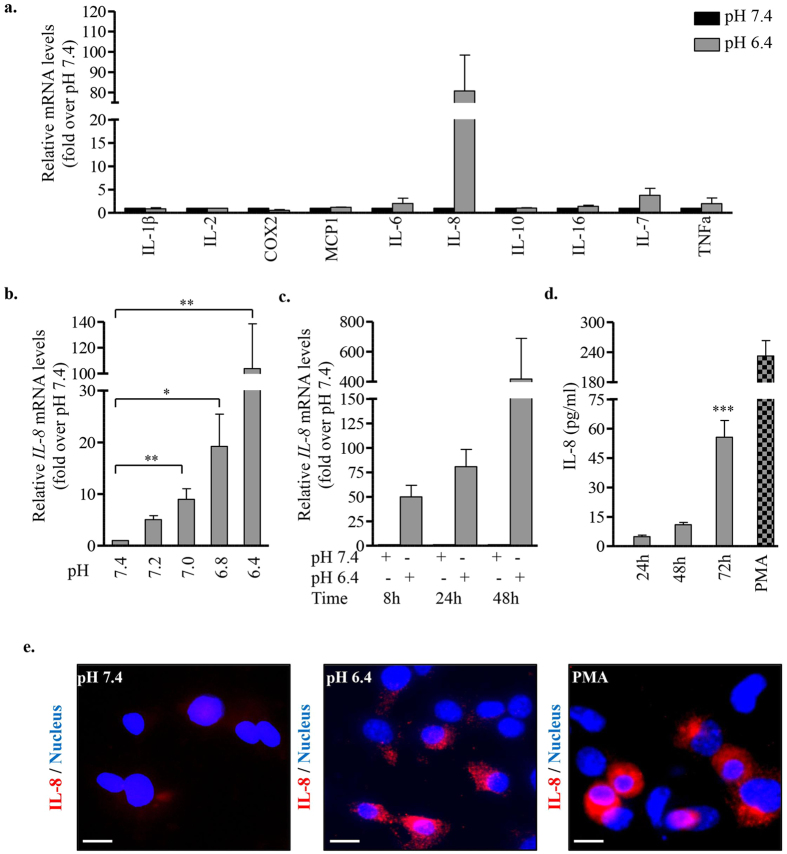
Acidic extracellular pH induces the expression and secretion of IL-8 by EndoC-βH2 cells. (**a**) EndoC-βH2 cells were cultured at pH 7.4 or 6.4 for 24 h and screened by RT-qPCR for the expression of selected pro/anti-inflammatory cytokines. (**b**) *IL-8* expression was determined by RT-qPCR in EndoC-βH2 cells cultured at different pH for 24 h (one-way ANOVA, post test for linear trend (*p* = 0.0008)). (**c**) *IL-8* expression was determined by RT-qPCR in EndoC-βH2 cells cultured at pH 7.4 or 6.4 for the indicated time points (one-way ANOVA, post test for linear trend (*p* = 0.067)). (**d**) EndoC-βH2 cells were cultured at pH 7.4 or 6.4 for 24 h/48 h/72 h or with PMA (100 ng/ml for 12 h) (one-way ANOVA). Secreted IL-8 was quantified by ELISA. (**e**) Immunostaining for IL-8 in EndoC-βH2 cells cultured at pH 7.4 or 6.4 for 72 h or with PMA (100 ng/ml) for 12 h. Scale bar 10 μm. Data are mean ± SEM of 3–5 experiments. *p < 0.05; **p < 0.01; ***p < 0.001.

Extracellular acidic microenvironment has been reported to induce the expression of pro- and anti-inflammatory cytokines in a variety of cell types^3^,^28^. We examined the expression of selected cyto/chemokines by EndoC-βH2 cells exposed to acidic pH. RT-qPCR analysis of cells incubated at pH 7.4 or pH 6.4 for 24 h showed the induction of *IL-8* transcripts in cells exposed to acidic pH (Fig. 4a). At all intermediate pH tested between 7.4 and 6.4, IL-8 mRNA expression increased while pH decreased (Fig. 4b). Low pH-induced IL-8 mRNA expression was detected as early as 8 h following low pH exposition and increased at later time points (24 and 48 h) (Fig. 4c). Following incubation at pH 6.4, IL-8 protein was detected in the conditioned medium of EndoC-βH2 cells. PMA, a strong inducer of IL-8 in human EndoC-βH2 cell model, was used as positive inducer (Fig. 4d). pH dependent accumulation of IL-8 in EndoC-βH2 cells was further confirmed by immunostaining (Fig. 4e).

**Figure 5:**
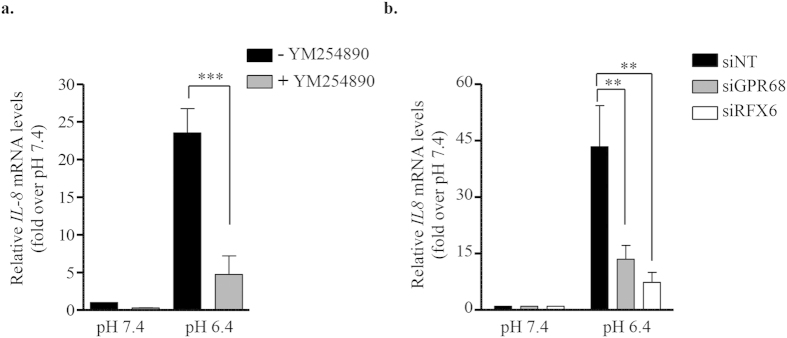
IL-8 induction upon extracellular acidification is RFX6- and GPR68-dependent. (**a**) EndoC-βH2 cells were cultured for 24 h at pH 7.4 or 6.4 with or without YM-254890 (100 nM), a G_q/11_-selective inhibitor compound. *IL-8* expression was analyzed by RT-qPCR. (**b**) EndoC-βH2 cells were transfected with siRNA (siNT), siGPR68 or siRFX6. After 48 h, cells were cultured for an additional 24 h period at pH 7.4 or 6.4 and next analyzed for the expression of *IL-8* by RT-qPCR. Data are mean ± SEM of 3–5 experiments. **p < 0.01; ***p < 0.001 (one-way ANOVA, followed by a Tukey’s multiple comparisons post-test).

To test whether acidification induced *IL-8* mRNA expression is mediated through GPR68, EndoC-βH2 cells were first incubated at acidic pH in the presence of the selective G_q/11_ inhibitor YM-254890. Under these conditions, the induction of *IL-8* mRNA upon acidic pH is dramatically dampened (Fig. 5a). Moreover both GPR68 siRNA and RFX6 siRNA also decreased acidic pH-induced *IL-8* mRNA induction (Fig. 5b).

### IL-8 induction in β-cells by extracellular acidification is NF-кB -dependent

**Figure 6:**
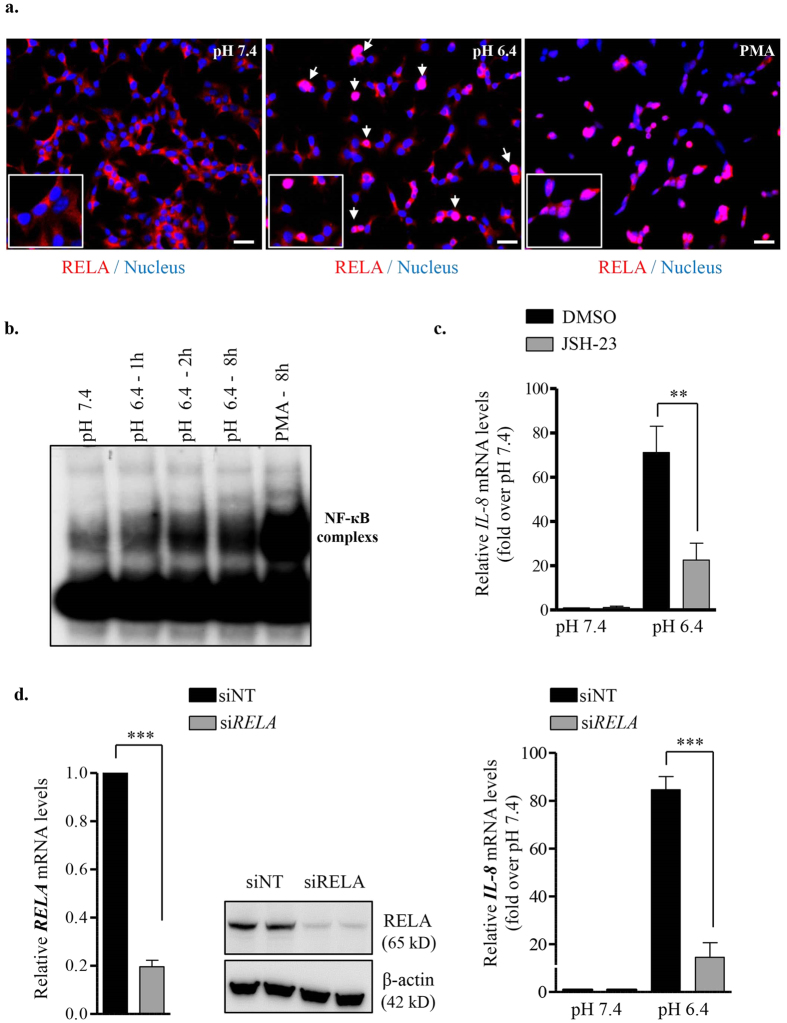
IL-8 induction in EndoC-βH2 cells by extracellular acidification is NF-кB dependent. (**a**) EndoC-βH2 cells were cultured at pH 7.4 or 6.4 for 12 h or with PMA (100 ng/ml for 8 h) and analyzed by immunofluoresence for the nuclear accumulation of RELA (p65) component of NF-кB complex. Scale bar 10 μm. (**b**) EndoC-βH2 cells were cultured at pH 7.4 for 8 h, at pH 6.4 for 1, 2 and 8 h or with PMA (100 ng/ml for 8 h). Proteins were extracted and used for Electrophoretic mobility shift assay (EMSA) analysis using a [^32^P] radiolabeled NF-кB probe. (**c**) EndoC-βH2 cells were treated with 1 μM NF-кB Activation inhibitor-II, JSH-23 for 24 h and analyzed for *IL-8* expression by RT-qPCR (one-way ANOVA). (**d**) EndoC-βH2 cells were transfected with either control siRNA (siNT) or siRNA targeted *RELA* (siRELA). After 48 h, siNT or siRELA transfected cells were cultured for 24 h at pH 7.4 or 6.4. Efficient RELA knock-down was verified by RT-qPCR and immunoblot analysis (left panel). The effect of pH on *IL-8* expression following RELA was quantified by RT-qPCR (right panel) (one-way ANOVA). Data are mean ± SEM of 3–5 experiments. **p < 0.01 and ***p < 0.001.

NF-кB is a central mediator of inflammatory response^29^ and RELA, a major subunit of NF-кB complex, is a mediator of IL-8 transcription^30^. As EndoC-βH2 cells secrete inflammatory cytokine IL-8 in response to acidification of their medium, we investigated whether IL-8 production requires NF-кB activation. We showed nuclear translocation of RELA, a subunit of NF-кB complex upon acidic pH treatment in EndoC-βH2 cells (Fig. 6a). EMSA performed using cellular extracts from EndoC-βH2 showed that DNA-binding activity of NF-кB increased in a time-dependent manner when cells were exposed to acidic pH 6.4 (Fig. 6b). Consistent with these results, cell treatment with JSH-23, a potent NF-кB activation inhibitor II, significantly decreased acidic pH induced *IL-8* mRNA expression (Fig. 6c). Moreover, a siRNA that efficiently targeted *RELA* (Fig. 6d, left), decreased the acidic pH-mediated *IL-8* mRNA induction (Fig. 6d, right). Thus, in EndoC-βH2 cells low pH induces the up-regulation of *IL-8* mRNA through the activation of NF-кB complex.

### IL-8 secreted by human β-cells in acidic conditions attracts neutrophils

**Figure 7:**
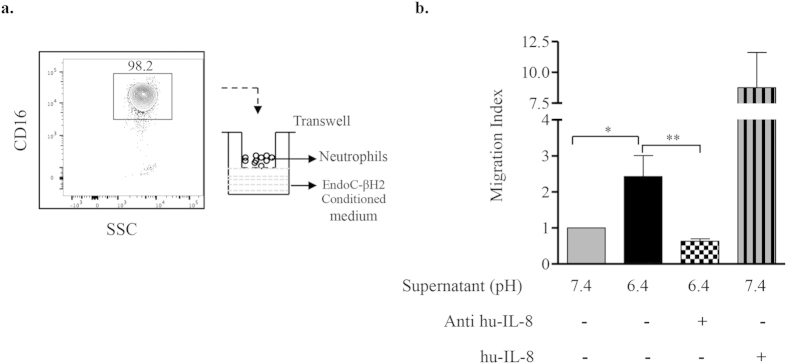
IL-8 secreted by EndoC-βH2 cells in acidic conditions attracts neutrophils. (**a**) Neutrophils were isolated from whole blood using MACSxpress kit, analyzed for the expression of CD16 and used for *in-vitro* transwell migration assay. (**b**) Neutrophil chemotaxis was tested using conditioned media from EndoC-βH2 cells cultured at pH 7.4 or pH 6.4 for 72 h. Acidic pH 6.4 conditioned medium pre-treated for 10 min with Anti-human IL-8 (1 μg/ml) was also used as well as pH 7.4 conditioned medium supplemented with recombinant human-IL-8 (50 ng/ml). Data are represented as migration index, calculated by assigning a value of 1 to the number of migrating neutrophils towards pH 7.4-conditioned medium. Data are mean ± SEM of 4 blood donors. *p < 0.05; **p < 0.01 (one-way ANOVA, followed by a Tukey’s multiple comparisons post-test).

IL-8 is a chemotactic pro-inflammatory cytokine that mediates the recruitment and activation of neutrophils during inflammation^31^. We examined if IL-8 secreted by EndoC-βH2 cells exposed to acidic pH induces neutrophil chemotaxis. We performed *in-vitro* migration assay using CD16-positive human blood neutrophils and EndoC-βH2 cell conditioned media as chemo-attractant (Fig. 7a). When compared to pH7.4 condition medium, acidic conditioned medium significantly attracted blood neutrophils. This chemotactic migration was abrogated by anti-IL-8 antibody indicating that neutrophil migration to acidic conditioned medium is dependent on the presence of IL-8 (Fig. 7b).

## Discussion

We and others recently showed that in adult human and mouse pancreatic β-cells, the transcription factor RFX6 controls insulin secretion by modulating calcium homeostasis^23^,^32^. In the present study, we demonstrate that RFX6 plays a pivotal role in extracellular proton sensing by regulating the expression of the G-protein coupled receptor GPR68 in human β-cells. We next demonstrate that extracellular signal acidification activates GPR68 which induces the production of inflammatory chemokine IL-8 through activation of the NF-кB complex.

GPR68 is one of the 4 known proton sensing GPCRs together with GPR4, 65 and 132^5^,^8^. Our results indicate that GPR68 is the predominant GPCR of this family in the human β cell-line EndoC-βH2 cells. This appears to be also the case in primary human β-cells. Indeed, human islet preparations that contain β-cells, but also some endocrine and non endocrine pancreatic cells, express GPR68 but no GPR65 and GPR132 (our present data and ref. 25). GPR4 expression is also detected in human islet preparations. However, while GPR68 remains expressed in islet β-cell enriched fractions, GPR4 expression levels collapse in such fraction^25^. Such data suggest that GPR68 is expressed in primary human β-cells, while GPR4 is expressed in non β-cells in islet preparations.

Signaling via GPCR plays major role in response to neurotransmitters, hormones and environmental stimulants^33^,^34^. This is also the case in pancreatic β-cells, where signaling mediated by a number of GPCRs regulate β-cell expansion and function^35^,^36^,^37^. However, limited information is available on the regulation of GPCR expression^38^,^39^. Here, using gain- and loss-of-function approaches, we provide strong evidence that RFX6 is a major positive regulator of GPR68 expression. RFX6 knockdown down-regulates GPR68 in EndoC-βH2 cells and in human islets. Likewise, overexpression of wtRFX6 enhances *GPR68* expression. Finally, converting RFX6 into a constitutive transcriptional activator (VP16-RFX6) or repressor (KRAB-RFX6) leads to chimeric proteins able to increase or decrease, respectively, *GPR68* expression. This regulation could be mediated by the direct binding of RFX6 to the X-box motifs on −1 K proximal promoter of GPR68 as revealed by promoter analysis using MatInspector (data not shown).

Previous data indicated that glucose-stimulated insulin secretion is attenuated in GPR68-deficient mice, suggesting that rodent β-cells sense protons via GPR68^22^. Here, we show that in human β-cells, GPR68 is expressed and is functional. Specifically, in human β-cells, extracellular acidification, through proton-sensing GPR68- G_q/11_ receptor stimulates IP production and activates NF-кB, giving rise to the production and secretion of the inflammatory chemokine IL-8. This last point couldn’t have been observed in rodent models, as IL-8 is not expressed by rodent cells and considered as a dead gene in murine genome^40^. Of note, the expression of *CXCL1*, the functional homologs of *IL-8* in rodents^41^, is not induced at low pH in the rodent β-cell line MIN6 (our unpublished data). This highlights the need to use human β-cell models such as EndoC-βH2 cells, limits the use of murine models and further confirms the major differences between rodent and human β-cells^42^,^43^.

In physiological conditions, interstitial pancreatic pH is maintained at ~7.4 while it drops to 7.0 or even below in a number of patho-physiological conditions. This is the case in chronic pancreatitis^44^, or in pancreatic adenocarcinoma^45^,^46^,^47^,^48^. Extracellular pancreatic acidification is also observed in T1D patients with ketoacidosis^15^,^16^. Whether and how low extracellular pH acts on human β-cells remains poorly studied. Here, we demonstrate that in human beta cells, GPR68 senses protons and signals by activating NF-кB complexes, resulting in IL-8 production and secretion. Interestingly, accumulating evidence indicate elevated levels of IL-8 in pancreas-related pathologies such as chronic pancreatitis^49^, pancreatic adenocarcinoma samples^50^,^51^ but also T1D patients with severe ketoacidosis episodes^52^. In this last case, IL-8, by acting as a chemo-attractant, would recruit neutrophils for further inflammatory responses and human β-cell destruction. In T1D, β-cells are destroyed by an auto-immune reaction and it had been thought that no β-cells can resists this aggression. However, recent data indicate that in subgroups of T1D patients, insulin-secreting β-cells that escaped destruction, remain present years after diagnostic^53^,^54^. A hypothesis would be that such patients had not developed ketoacidosis- induced IL-8 production.

In conclusion, we have identified RFX6 as an important transcriptional regulator of the proton-sensing receptor GRP68 in adult human β-cells. We also demonstrated that through GPR68/_Gq/11_ and NF-кB activation, extracellular acidification induces inflammatory chemokine IL-8 production. Earlier reports demonstrated the effectiveness of GPR4 antagonist to block inflammatory response to acidosis in endothelial cells^55^,^56^. Moreover very recent reports have characterized specific modulators of GPR68^57^. In this context, identifying GPR68 antagonists could be novel therapeutic targets for the improvement of inflammatory conditions associated with pancreatic acidosis.

## Methods

### Culture of human cell lines and islets

EndoC-βH2 cells^24^ were cultured in low-glucose (1g/L) Dulbecco’s modified Eagle’s medium (DMEM; Sigma-Aldrich) containing L-glutamine and sodium pyruvate, supplemented with 2% BSA fraction V (Roche Diagnostics), 50 μM 2-mercaptoethanol, 10 mM nicotinamide (Calbiochem), 5.5 μg/ml transferrin (Sigma-Aldrich), 6.7 ng/ml selenite (Sigma-Aldrich), 100 U/ml penicillin and 100 μg/ml streptomycin. Cells were seeded on Matrigel (1%)/fibronectin (2 μg/ml) (Sigma-Aldrich) coated plates and cultured at 37 °C and 5% CO_2_. The human duct cell line SKPC^58^ was cultured in high glucose DMEM (4.5 g/L) supplemented with 10% fetal calf serum (Biowest), 100 U/ml penicillin and 100 μg/ml streptomycin. Human islets were isolated and maintained as described^59^.

### Preparation of buffered culture media

Culture media at different pH were prepared as described^8^,^60^. Briefly, low glucose DMEM without sodium bicarbonate (Sigma, D2902) was buffered with HEPES (4-(2-Hydroxyethyl) piperazine-1-ethanesulfonic acid; Sigma, H0887), EPPS (4-(2-Hydroxyethyl)-1-piperazinepropanesulfonic acid; Sigma, E9502) and MES (2-(N-Morpholino) ethanesulfonic acid; Sigma, M3671) (8mM each) and pH was adjusted with HCl/NaOH. They were kept iso-osmotic by adding NaCl and NaHCO_3_. The pH stability was monitored at the initiation and completion of the experiments.

### RNA isolation, reverse transcription and RT-qPCR

Total RNA was extracted from EndoC-βH2 cells using RNeasy Plus Micro kit (Qiagen). First strand cDNA was prepared using Maxima First Strand cDNA synthesis kit (ThermoFisher). RT-qPCR was performed using Power SYBR Green mix (Applied Biosystems) with ABI Prism 7300 sequence detector (Applied Biosystems). *Cyclophilin A* transcript levels were used for normalization of each target gene. The custom primers were designed with IDT Primer-Quest online software and the amplification efficiency for each primer was determined with serial dilution of total cDNA from EndoC-βH2/human islets cDNA. Primer sequences are listed in Supplementary Table.

### siRNA Transfection

EndoC-βH2 cells were transfected using Lipofectamin RNAiMAX (life technologies) and ON-TARGET*plus* siRNA SMARTpool for human *RFX6*/*GPR68*/*RELA* gene (40 nM) or ON-TARGET*plus* Non-targeting pool (siNT) (Dharmacon, Thermo Scientific) as described^23^. Human islet samples were partially dissociated with Accutase (PAA Laboratories) and siRNA transfections were performed as described^23^.

### DNA Transfection

Human RFX6 constructs (pRIG-RFX6, pRIG-Mut506RFX6, pRIG-KRAB-RFX6 and pRIG-VP16-RFX6)^23^ were used in this study. The MGC Human GPR68 cDNAclone (Clone ID: 6971805) was purchased (Open Biosystems; Thermo Scientific) and sub-cloned into pEGFP-N1 (Clonetech). EndoC-βH2 cells were transiently transfected with DNA using Lipofectamin2000 (Invitrogen) following manufacturer’s instructions in Opti-MEM. GFP-positive cells were FACS sorted 24–48 h post transfections and RNA expression was analyzed by RT-qPCR.

### Electrophoretic mobility shift assay for NF-kB

EndoC-βH2 cells were cultured at pH 7.4 (8 h) or 6.4 (1, 2 and 8 h) or with PMA (100 ng/ml at pH 7.4 for 8 h). Cellular extracts were prepared and NF-кB activation was analyzed by electrophoretic mobility shift assay (EMSA) using the human immunodeficiency virus long terminal repeat tandem кB oligonucleotide as кB probe^61^.

### IP and cAMP formation assay

IP formation was quantified with HTRF (Homogeneous Time-Resolved Fluorescence) based “Cisbio IP-One Tb” (Cisbio, Bagnols-sur-Cèze, France) assay kit, following manufacturer’s instructions. EndoC-βH2 cell suspensions (5 × 10^4^ cells) were treated in 384-well plate (16 μl volume) with modified stimulation buffer (10 mM Hepes, 10 mM MES, 1 mM CaCl2, 0.5 mM MgCl_2_, 4.2 mM KCl, 146 mM NaCl, 5.5 mM glucose, 50 mM LiCl) at pH 7.4 or 6.4 for 60 min at 37 °C. Where indicated, cells were pretreated with YM-254890, a selective Gα_q/11_ inhibitor^27^ for 30 min prior to incubation with the IP stimulation buffer and maintained throughout the IP determination. IP measurements were performed in triplicates and experiments were repeated at least three times. Samples were read on a *TECAN* Infinite F500 (Tecan Group, Ltd., Männedorf, Switzerland) with excitation at 320 nm and emission at both 620 nm and 665 nm.

cAMP activity was measured using a cAMP-HTRF assay kit (Cisbio) following manufacturer’s instructions. EndoC-βH2 cells (5 × 10^3^) were treated in 384-well plate (12 μl final volume) with stimulation buffer (PBS containing 10 mM of each HEPES, MES, and 0.5 mM IBMX, at pH 7.4 or 6.4) for 30 min at room temperature. Cells were lysed using kit lysis buffer and cAMP was then measured in 384 well plates (HTRF) with *TECAN* Infinite F500.

### Immunocytochemistry and immunoblotting

EndoC-βH2 cells were cultured on Matrigel/fibronectin coated 4-well chambers slide (Nunc Lab-Tek) and processed for IL-8 and RELA immunostaining as described^62^ using anti-IL-8 (1:1,000; BD554717; BD Biosciences) or anti-RELA (1:200; sc8008; Santa Cruz Biotechnology) antibodies. Images were acquired with a Leica Leitz fluorescent microscope equipped with cooled 3-chip charge coupled device camera (Hamamatsu C5810; Hamamatsu) and processed using ImageJ software.

For immunoblot assays, total cellular proteins were prepared as described^23^. Proteins (25 μg) were resolved by SDS PAGE, immunoblotted with antibodies against RELA (sc8008, 1/250 dilution) and Actin (1/1000, Sigma-Aldrich). Membranes were incubated with species-specific HRP-linked secondary antibodies (1:5000) and visualization was performed following ECL exposure.

### IL-8 Elisa

Secreted IL-8 protein levels were determined using commercially available Human IL-8 ELISA MAX Deluxe kit (BioLegend #431504) as per manufacturer’s instructions. EndoC-βH2 cells were treated with pH 7.4 or 6.4 or with 100 ng/ml PMA (at pH7.4) and culture supernatants were collected and stored for ELISA.

### Neutrophil migration assay

Blood samples were obtained from the pediatric endocrinology and diabetes center at Necker Enfants-Malades hospital, Paris, France in accordance with the approved guidelines. All the experimental protocols were approved by the local ethic committee (CPP - Paris Ile de France, France). Informed consent was obtained from all subjects. Neutrophils were isolated using a MACSxpress human neutrophil isolation kit (Miltenyi Biotec). Red Blood Cell lysis buffer was used to remove residual erythrocytes. The purity of isolated neutrophils was consistently between 98–99% based on CD16 staining. Chemotaxis assay was performed in 24-well micro chemotaxis chamber using 6.5 mm Transwell with 3 μm PVP-free polycarbonate filter membrane (Costar). Neutrophils (2 × 10^5 ^cells in 200 μl PBS) in upper chamber were allowed to migrate towards 500 μl of conditioned medium produced during 72 h by EndoC-βH2 cultured at pH 7.4 or 6.4. In some experiments, conditioned medium was supplemented with Anti-human IL-8 (1 μg/ml for 10 min; BD554717; BD Bioscience) or with recombinant human-IL-8 (50 ng/ml; BioLegend). After 2 h at 37 °C, migrating cells were recovered with Accutase (Sigma) in the lower chamber and numbered by flow cytometry. Results are expressed as migration index: number of migrating neutrophils in a defined condition divided by number of migrating neutrophils towards pH 7.4 conditioned medium.

### Statistics

Graphs were constructed by using PRISM software (version 5.02 GraphPad). Quantitative data are presented as the mean ± SEM from at least three independent experiments, unless indicated. For comparison between two mean values, statistical significances were estimated using two-tailed Student’s *t*-test. For comparison between three or more values, one-way ANOVA was used with Tukey’s multiple comparisons post-test. Statistical significance was set at p < 0.05.

## Additional Information

**How to cite this article**: Chandra, V. *et al*. Extracellular acidification stimulates GPR68 mediated IL-8 production in human pancreatic β cells. *Sci. Rep.* **6**, 25765; doi: 10.1038/srep25765 (2016).

## Supplementary Material

Supplementary Information
