# Supplementary material for: Extracellular acidification stimulates GPR68 mediated IL-8 production in human pancreatic β cells
Source: Sci Rep. 2016 May 11;6:25765. doi: 10.1038/srep25765 (PMC4863151; doi:10.1038/srep25765)
Supplement: Supplementary Information [file srep25765-s1.pdf]

Supplementary Information

Extracellular acidification stimulates GPR68 mediated IL-8 production in human pancreatic β cells

Vikash Chandra, Angeliki Karamitri, Paul Richards, Françoise Cormier, Cyrille Ramond, Ralf Jockers, Mathieu Armanet, Olivier Albagli-Curiel, Raphael Scharfmann

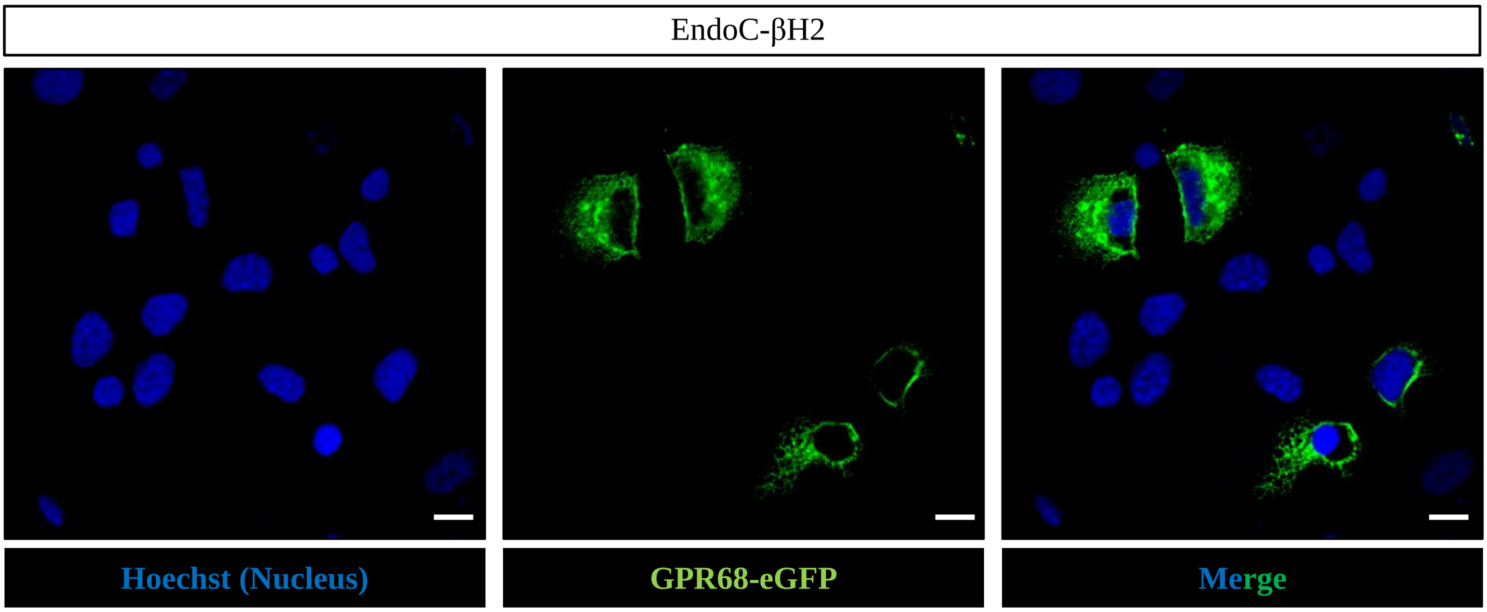

Supplementary Figure 1.

Localization of GPR68-EGFP protein ectopically overexpressed in EndoC-βH2.

EndoC-βH2 cells were transiently transfected with CMV-GPR68-EGFP construct and Cells were alayzed by fluorescencent microscopy 24h post-transfection. Scale bar 10μm.

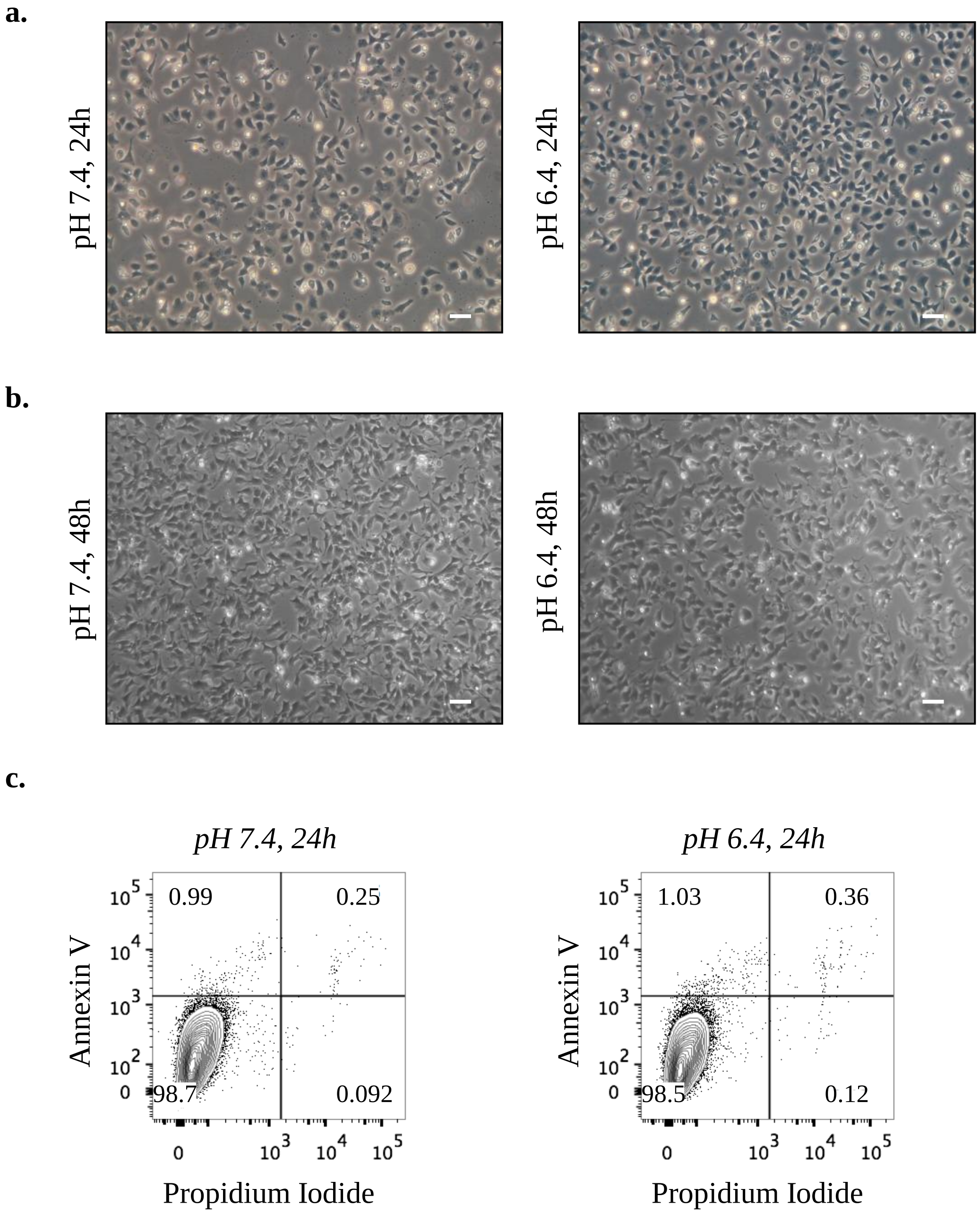

Supplementary Figure 2.

Extracellular acidic pH does not affect EndoC-βH2 cells viability.

(a-b) EndoC-βH2 cells were cultured at pH 7.4 or 6.4 and phase contrast images were taken after 24h and 48h. (**c**) EndoC-βH2 cells were treated with pH 7.4 or 6.4 for 24h and cell viability was measured by AnnexinV^{FITC}/PI staining (n=3). Scale bar 100µm.

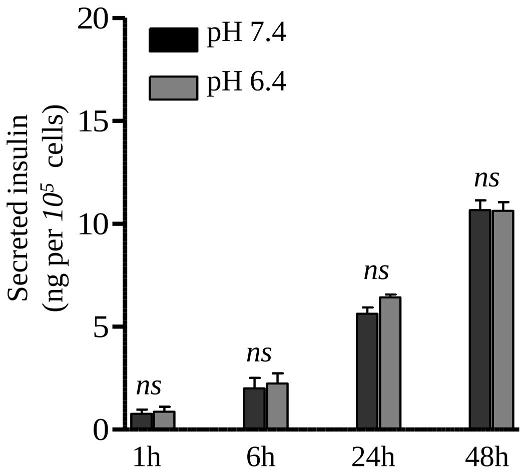

Supplementary Figure 3.

Effect of extracellular acidification on insulin secretion.

EndoC-βH2 cells were cultured at pH 7.4 or 6.4. Culture supernatants were collected after 1h, 6h, 24h and 48h and secreted insulin was measured. *ns*, non-significant (one-way ANOVA, followed by a Tukey's multiple comparisons post-test).

Supplementary Table: List of primers used in this study for RT-qPCR.

Gene (Human)	**Forward primer (5' – 3')**	**Reverse Primer (5' – 3')**
RFX6	CATGTCGAACTCCAGTCCTAGCTT	AGTCCAGGGTTTCTTGAGCTGGAT
GPR68	AGGTCATCGAGGACGAGAA	CAGGAAGCGGTAGTAGTTGATG
GPR4	GTTGACACACTGACTCCATACA	GGAGGGATGGAATTATGACAGG
GPR65	CAGCATGACCTGGATCACTATT	GCAGGAAAGACACACACAGA
GPR132	GGTGGTTGTCATCTTCCTAGTC	CGTTCCTGTCTCCTCTGTAGTA
IL-1β	CTCTCACCTCTCCTACTCACTT	TCAGAATGTGGGAGCGAATG
IL-2	CTCACCAGGATGCTCACATTTA	CCTCCAGAGGTTTGAGTTCTTC
IL-8	AAATCTGGCAACCCTAGTCTG	GTGAGGTAAGATGGTGGCTAAT
IL-6	CCAGAGCTGTGCAGATGAGT	GGGTCAGGGGTGGTTATTGC
COX2	TACTGGAAGCCAAGCACTTT	GGACAGCCCTTCACGTTATT
IL-7	GTGTCGTCCGCTTCCAATAA	ACTGGGAGCTAGAACCATGA
IL-10	TTTCCCTGACCTCCCTCTAA	CGAGACACTGGAAGGTGAATTA
IL-16	AGTCTGGTCAGTCCGTTATCT	GTGACATGGATGCCGTCTAAT
TNFa	GATCCCTGACATCTGGAATCTG	GAAACATCTGGAGAGAGGAAGG
MCP1	TCATAGCAGCCACCTTCATTC	CTCTGCACTGAGATCTTCCTATTG
RELA	TGAGCCCACAAAGCCTTATC	ACAATGCCAGTGCCATACA
Cyclophilin	ATGGCAAATGCTGGACCCAACA	ACATGCTTGCCATCCAACCACT
(Mouse)		
CXCL1	GCTGGGATTCACCTCAAGAA	TGGCTATGACTTCGGTTTGG

Supplementary Experimental Procedures

GPR68-EGFP construct and transient transfection

The MGC Human GPR68 cDNAclone (Clone ID: 6971805) was purchased (Open Biosystems; Thermo Scientific) and sub-cloned into pCDNA3.1NEGFP. EndoC-βH2 cells were transiently transfected with DNA using Lipofectamin2000 (Invitrogen) following manufacturer's instructions in Opti-MEM as described above.

Annexin V-PI staining

Endoc-bH2 cells were treated with pH 7.4 or 6.4 for the indicated time-point and apoptosis were determined by using BD Annexin V: FITC apoptosis detection kit (BD Bioscience #556547) as per manufacturer instructions.

Insulin Secretion

EndoC-βH2 cells were treated with pH 7.4 or 6.4. At different time points, supernatant was collected and stored at -20°C until insulin ELISA assay. Insulin secretions were measured by using a commercial human insulin ELISA kit (Mercodia) according to the manufacturer's instructions.
